# Scalable Epitaxial Growth of WSe_2_ Thin Films on SiO_2_/Si *via* a Self-Assembled PtSe_2_ Buffer Layer

**DOI:** 10.1038/s41598-019-44518-3

**Published:** 2019-05-29

**Authors:** Pei-Chen Wu, Chun-Liang Yang, Yuanmin Du, Chih-Huang Lai

**Affiliations:** Department of Materials Science and Engineering, National Tsing Hua University, 30013 Hsinchu, Taiwan

**Keywords:** Synthesis and processing, Electronic devices

## Abstract

The growth of large-area epitaxial transition metal dichalgogenides (TMDCs) are of central importance for scalable integrated device applications. Different methods have been developed to achieve large-sized high quality films. However, reliable approaches for centimeter-sized or even wafer-level epitaxial growth of TMDCs are still lacking. Here we demonstrate a new method to grow inch-sized epitaxial WSe_2_ films on SiO_2_/Si substrates at a much lower temperature with high repeatability and scalability. High quality crystalline films are achieved through direct selenization of a tungsten film with platinum as the underlayer. The self-assembled PtSe_2_ buffer layer, formed during selenization, assists epitaxial growth of WSe_2_. Using fabricated WSe_2_ films, excellent performance memory devices are demonstrated. As a member of the TMDC family, our findings based on WSe_2_ may also be applied to other TMDC materials for large-scale production of high quality TMDC films for various applications.

## Introduction

Owing to the strong in-plane covalent bonds and weak van der Waals bonding between adjacent layers, layered materials have been extensively studied in the past decade due to their attractive properties^1–4^. As two-dimensional inorganic analogues of graphene, transition metal dichalgogenides (TMDCs) with the formula MX_2_, where M is a transition metal atom and X is a chalcogen atom, have shown great potential for nanoelectronic device applications such as transistors^5–8^, optoelectronic devices^9–12^, and energy storage devices^13,14^. With a lattice structure similar to well-known MoS_2_, WSe_2_ is another interesting TMDC material. Although most of the applications require TMDC thin films with large area and good quality, the large-scale production of high quality TMDC films is still challenging.

Similar to other TMDC materials, chemical vapor deposition (CVD) is a frequently used method for WSe_2_ synthesis^15–18^. Although CVD growth of micrometer-sized 2D WSe_2_ structures has been well developed, it typically requires high temperature process (700 to 1300 °C) and also has the limits of the substrates used for the synthesis^16–19^. Due to large surface energy and poor wettability to the insulating substrates, the formation of WSe_2_ tends to aggregate into amorphous structures instead of highly crystalline crystals^19,20^. Large-area CVD prepared WSe_2_ films on glass substrates at a lower temperature from reaction of WCl_6_ and diethyl diselenide have been reported^15^, but they are composed of either platelet or needle like crystalline structure with different orientations. Through a vapor transfer method^21,22^, highly textured WSe_2_ films have been fabricated on insulating substrates, however, films with high crystallinity and scalability are still lacking. Inspired by the selenization method widely used in the fabrication of Cu(In_1−x_Ga_x_)Se_2_ (CIGS) solar cells^23,24^, here we report a new method for large-area epitaxial WSe_2_ synthesis on SiO_2_/Si substrates. In contrast to the widely used method of synthesis *via* selenization of WO_3_, we demonstrate a direct approach to form WSe_2_ by selenizing W with the assistance of a Pt underlayer. In the end, a mechanism involved with the formation of a self-assembled PtSe_2_ buffer layer has also been proposed.

## Results and Discussion

### Scalable epitaxial WSe_2_ film

**Figure 1:**
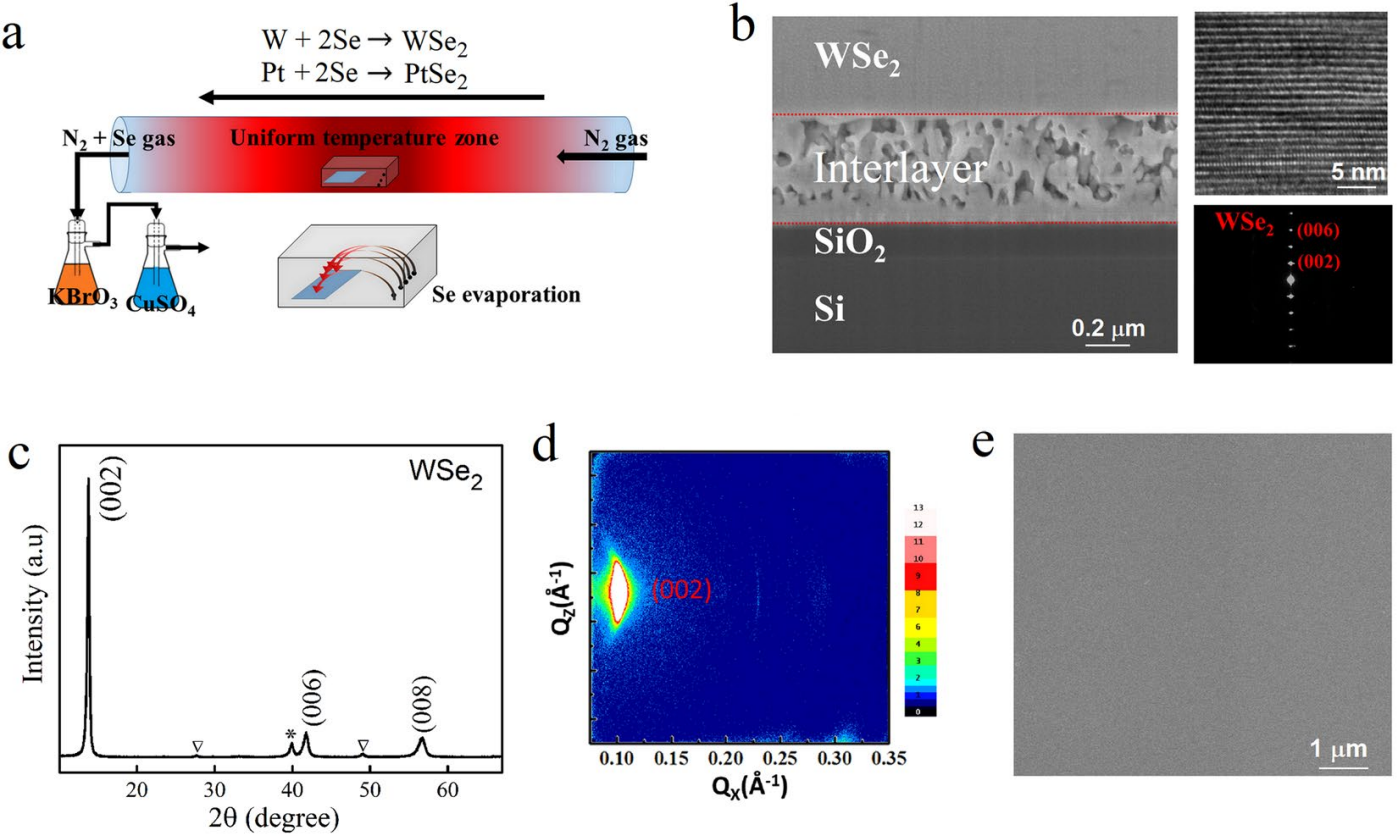
The epitaxial growth of WSe_2_. (**a**) The synthesis process of WSe_2_ with a W/Pt/Ta/SiO_2_/Si substrate through a two-step selenization process. (**b**) Cross-sectional SEM image of a FIB-prepared lamella. Right up is a TEM image for WSe_2_, and right below is the SAED pattern, confirming the [001] oriented WSe_2_ lattice structure. (**c**) XRD patterns of the stacked film. Highly [001] orientated WSe_2_ is shown. ∇ and * stand for peaks from PtSe_2_ and Pt_2_W, respectively. (**d**) 2D-GIXRD image. (**e**) SEM top-view image of the WSe_2_ film showing smooth surface morphology.

A schematic illustration for the growth of WSe_2_ is shown in Fig. 1a. To synthesize WSe_2_, an inch-sized (up to 40 × 40 mm^2^) SiO_2_/Si substrate is firstly coated with Pt 100 nm/Ta 3 nm by magnetron sputtering at room temperature. Ta works as an adhesion layer and also promotes the growth of Pt(111)^25^. A α-phase W(110) film is then sputtered on Pt (see Supplementary Fig. S1 for details), followed by a two-step selenization process in a furnace: a first step at 350 °C for the initial selenization and another step at 550 °C for the final treatment. Figure 1b (left) shows a cross-sectional scanning electron microscopy (SEM) image of the stacked film structure. The large-area highly epitaxial growth of WSe_2_ along the [001] direction has been achieved, depicted by the high-resolution transmission electron microscope (HRTEM) image and selected area electron diffraction (SAED) pattern (Fig. 1b, right). In addition, an interlayer with different morphology is observed below the WSe_2_ layer (Fig. 1b SEM image). The X-ray diffraction (XRD) patterns (Fig. 1c) reveal that peaks related to the WSe_2_ diffraction from high order (00ℓ) planes are observed. A strong WSe_2_ (002) peak at 2θ = 13.7° indicates high crystallinity of the film. In addition to the strong WSe_2_ peaks, weak XRD peaks of Pt_2_W and PtSe_2_ have also been observed. The elemental W/Se ratio in the WSe_2_ layer is close to 0.5, confirmed by energy dispersive X-ray spectroscopy (EDX) (see Supplementary Fig. S2 for details), indicating a good stoichiometry of the synthesized WSe_2_ film. To further reveal the in-plane crystalline property of the film, two-dimensional grazing incidence XRD (2D-GIXRD) is performed. As shown in Fig. 1d, a sharp and strong spot from WSe_2_ (002) is observed, indicating the high quality of the epitaxial film. Figure 1e shows a top-view SEM image of the WSe_2_ film. The fabricated WSe_2_ films have a smooth surface, which is generally required for large–scale device fabrications.

For comparison, we also use the same process to grow WSe_2_ directly on SiO_2_/Si substrates, that is, direct two-step selenization of a W layer without a Pt underlayer. The XRD patterns (Fig. S3a) reveal that the intensity of WSe_2_ (00ℓ) is much weaker for the sample without the Pt underlayer. The much poor crystalline quality for the WSe_2_ film directly grown on SiO_2_ is the same as the previous report^15,21^. In additon, unlike a smooth surface observed for the sample with Pt underlayer (Fig. 1e), the WSe_2_ film without the Pt underlayer shows quite rough surface with embedded nanoflake-like structures (Fig. S3b,c), similar to the rough surface morphology reported by earlier works for large-area WSe_2_ synthesis on dielectric substrates^15,21^.

**Figure 2:**
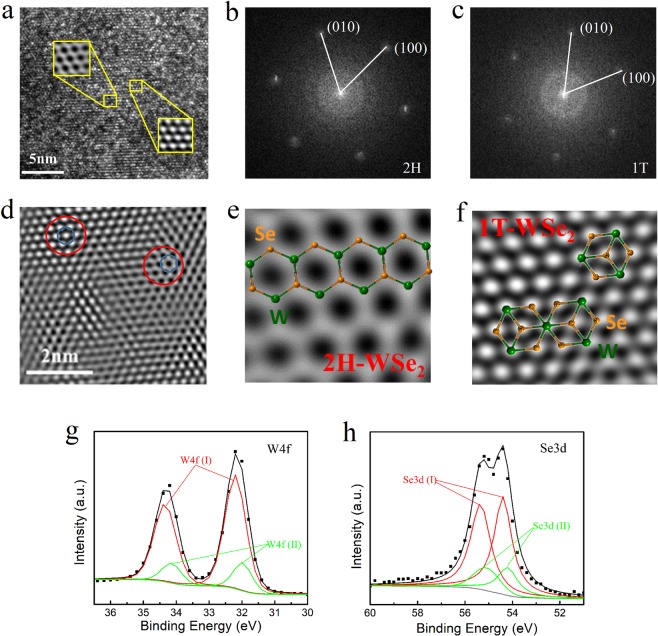
The co-existence of 2H and 1T WSe_2_ structures. (**a**) Top-view TEM image after a FIB cut of the sample. Two squared regions are enlarged to show the different contrast. (**b**,**c**) FFT images for the 2H and 1T phases respectively, and an offset of 30° is shown. (**d**) High resolution HAADF-STEM image. (**e**) Enlarged HAADF-STEM image showing 2H hexagonal structure of WSe_2_. (**f**) Enlarged HAADF-STEM image showing 1T hexagonal structure of WSe_2_. (**g**) XPS spectra of W 4f and (**h**) Se 3d. Core-level peaks of WSe_2_ from the 2H and 1T phases are shown in red and green colors, respectively.

To further investigate the structure of the layered WSe_2_ film grown by using a Pt underlayer, we perform a top-view high-angle annular dark-field scanning transmission electron microscopy (HAADF-STEM) analysis of the films obtained. Figure 2a illustrates the TEM image of a focuse ion beam (FIB) cut at the top region of the sample. It reveals a high crystalline quality of the film. Interestingly, a co-existence of 2H and 1T phases has been observed, as indicated by the enlarged images (inset of Fig. 2a). The hexagonal crystal structure of WSe_2_ is also evaluated from the fast Fourier transformation (FFT) image in Fig. 2b,c for two selected spots. An offset of 30° between the two sets of the hexagonal spots, identified as the 2H and 1T phases respectively, has been observed, similar to the earlier reports^26–28^. In the 2H phase, the atoms are in the hexagonal symmetry with trigonal prismatic coordination, while in the 1T phase an octahedtal manner is presented. Figure 2d shows high resolution image of that shown in Fig. 2a, with the mixing of both 2H and 1T structures. The atomic resolution images further demonstrate that the tungsten and selenium atoms are arranged in a hexagonal lattice configuration of WSe_2_, with both 2H (Fig. 2e) and 1T (Fig. 2f) structures. The high crystalline quality and co-existence of 2H and 1T are also confirmed in various regions of the sample (see Supplementary Figs S4 and S5 for more details).

X-ray photoemission spectroscopy (XPS) is conducted to acquire the binding energies of W 4f and Se 3d, as shown in Fig. 2g,h. The W 4f shows two large peaks at 34.3 eV and 32.2 eV (Group (I), color in red), attributed to the doublet W 4f_5/2_ and W 4f_7/2_, respectively. In addition, it also shows that there also exist two smaller peaks located at 34.1 eV and 32.0 eV (Group (II), color in green), a shift of the larger peaks. According to the earlier reports^29,30^, the Group (I) and Group (II) W 4f peaks can be attributed to 2H and 1T WSe_2_ phases respectively, which further supports the coexistence of 2H and 1T forms, consistent with the TEM image analysis. Similarly, it is also observed that the doublet Se 3d peaks, corresponding to the Se 3d_5/2_ and Se 3d_3/2_ orbital of divalent selenium ions (Se2^−^), can be separated into two groups (Fig. 2h). Compared to the semiconducting 2H phase, the 1T phase is known to be metallic and thermodynamically unstable. In our results, a stable 1T phase is consistently observed, which might come from the co-existence of its counterpart (2H phase).

**Figure 3:**
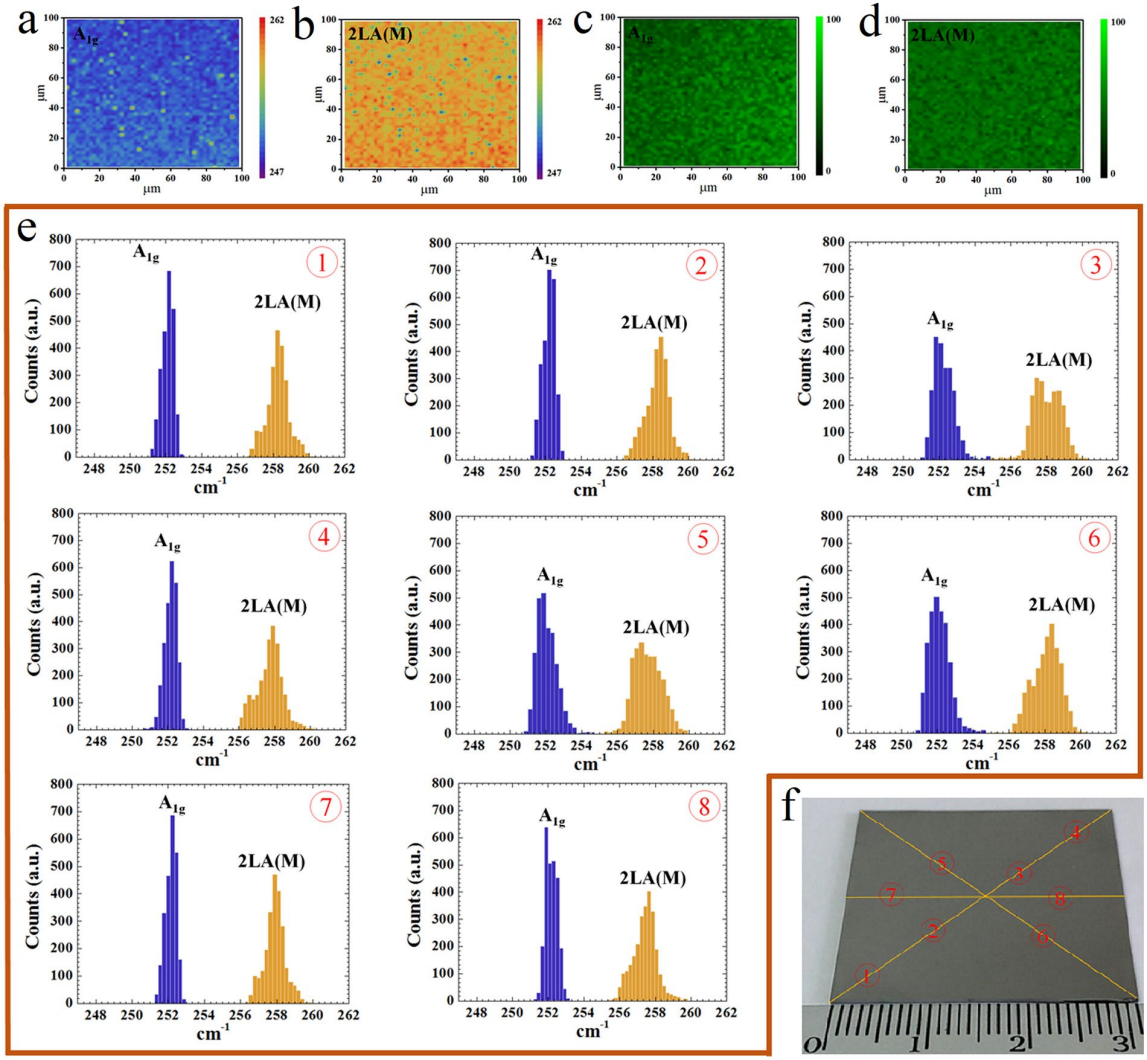
Characterizations of the macroscopic uniformity of the WSe_2_ film. (**a**,**b**) Raman spectra mappings of a 100 × 100 μm^2^ area of an as-deposited sample, representative to A_1g_ and 2LA(M) modes respectively. (**c**,**d**) Corresponding Raman intensity mappings of the two Raman characteristic A_1g_ and 2LA(M) modes, respectively. (**e**) Peak distributions for A_1g_ and 2LA(m) modes at different positions (100 × 100 μm^2^) on the sample surface. (**f**) Photograph of a WSe_2_ film on SiO_2_/Si wafer and the positions marked for Raman mappings.

We use the Raman mapping technique to explore the uniformity of the WSe_2_ film, across the inch-sized silicon substrate, based on the two characteristic A_1g_ and 2LA(M) modes^31,32^ in the region from 245 to 260 cm^−1^, as shown in Fig. 3. Figure 3a,b demonstrate the peak position mappings during the scans. Except for some outlier dots, a narrow variation of both peaks is obtained. In addition, Fig. 3c,d show the intensity mappings for the two peaks, supporting the uniform formation of epitaxial WSe_2_ films^33,34^. Through a surface mapping, no major difference can be found on the eight points (marked as ① to ⑧ in Fig. 3f) across the sample surface, as confirmed by the Raman peak position mappings in Fig. 3e.

**Figure 4:**
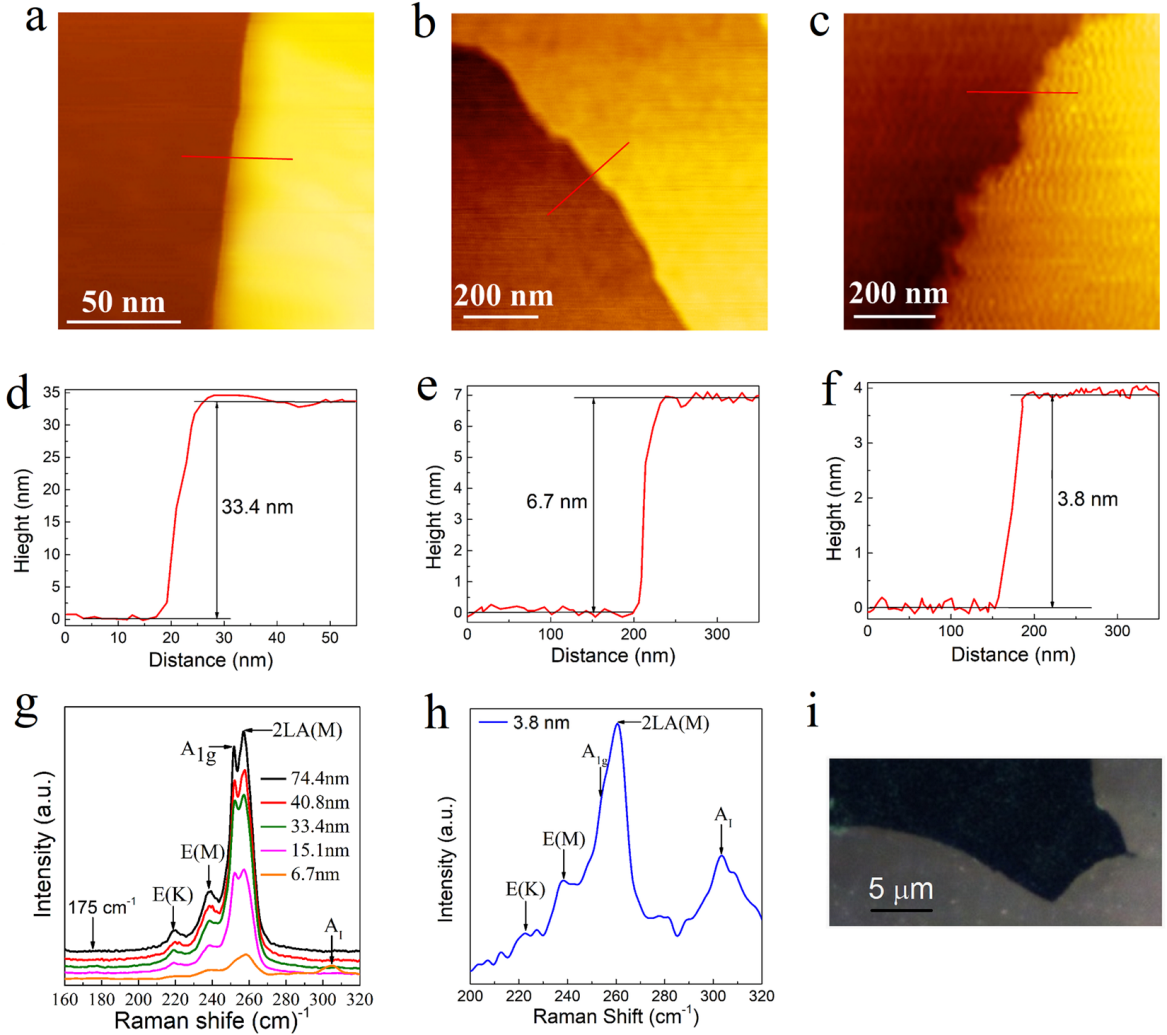
WSe_2_ flakes with different thickness after an exfoliation process. (**a**–**c**) AFM images, and (**e**,**f**) the corresponding height profiles (acquired on the area highlighted with a red line) for the WSe_2_ flakes. (**g**) Raman spectra of the exfoliated films with different thickness. 2LA(M), A_1g_, E(M), E(K) and A_I_ stand for different Raman modes for WSe_2_. 175 cm^−1^ refers to the position of the characteristic mode (E_g_) for PtSe_2_, and no peak is present. (**h**) Raman spectrum of WSe_2_ with 3.8 nm thickness. (**i**) Optical image of a WSe_2_ flake after a liquid exfoliation process.

CVD is the frequently used method to grow 2D material films on different substrates. But the development of optimized growth of single or multiple layers in a large scale has proven to have many difficulties. In addition to CVD, one common method for obtaining TMDC monolayers is mechanical exfoliation. Although its scale-up is limited, it is expected that the mechanical exfoliation method will continue to play an important role in the development of 2D materials. While different methods^35–39^ have been developed to improve the exfoliation process in the last decade, here we demonstrate the use of the original exfoliation (repeated peeling) method^35^ to get good quality of nanosheets from our layered WSe_2_ films. WSe_2_ nanosheets with different thickness after an exfoliation process are shown in Fig. 4. Figure 4a–c show the atomic force microscopy (AFM) images for the samples, with the thicknesses determined by the AFM measurements in Fig. 4d–f. Figure 4g shows the Raman spectra for WSe_2_ with different thickness. The 2LA(M), A_1g_, E(M) and E(K) are Raman modes corresponding to different scattering modes according to the report^31^. A good consistency of the Raman spectra has been shown for the exfoliated films. With the thickness going down to 15.1 nm, the A_I_ mode starts to appear^31^. Figure 4h shows the Raman spectrum of the 3.8 nm sample (~5 monolayers), for which a strong A_I_ mode has been shown. The appearance of the A_I_ band could be due to an interlayer vibration mode, which is reported to exist at a few layers^31,40^. The quality of the bulk crystal, and the van der Waals forces between the sheets of the layered structure determine the exfoliation process. Figure 4i shows one example of the exfoliated WSe_2_ flake after a liquid exfoliation process similar to the method described by Lin *et al*.^39^. The promising result achieved here means that we may get even larger areas of WSe_2_ 2D films, by using an improved method. We also need to mention that, through Raman analysis of the exfoliated films, no PtSe_2_ spectrum has been found, indicating high purity of the WSe_2_ films obtained.

**Figure 5:**
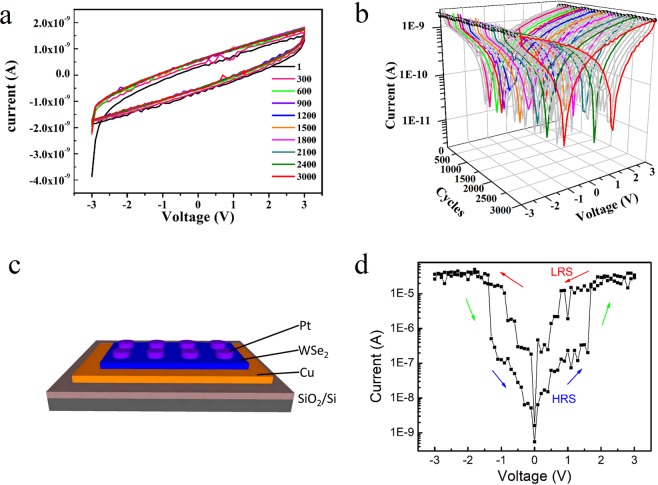
Device performance of two memory devices. (**a**) I-V curves for 3000 times of the as grown structure. Electrode size: 300 μm in dimension. (**b**) The I-V curves of (**a**) in semilogarithmic scale. Butterfly shaped curves are presented. (**c**) A ReRAM device structure composed of an exfoliated WSe_2_ nanosheet, with Pt the top electrode, and Cu the bottom electrode. (**d**) I-V characteristic of the device shown in (**c**).

To demonstrate and evaluate the electrical performance of the fabricated WSe_2_ films, stacked device structures are fabricated. Figure 5a,b show the typical current-voltage (I-V) characteristics of the device fabricated using the as grown WSe_2_ film, with Pt as the top electrode. A remarkable I-V performance has been shown, with high repeatability and reliability. It is a kind of capacitance memory effect, according to the report by Paul *et al*.^41^. Through the application of an external electrical field, charge storage and retention can be achieved. By utilizing the 2H-1T phase transition of MoS_2_ in a battery cell, high levels of pseudocapacitive charge storage have been reported by Cook *et al*. recently^42^. While the detailed mechanism needs to be further explored for our device structure, the highly repeatable result (more than 3000 cycles) indicates that the fabricated film has a good potential for battery material applications. Memory devices using an exfoliated WSe_2_ nanosheet are also fabricated. Figure 5c shows a resistive random access memory (ReRAM) device, through transferring a WSe_2_ nanosheet to a Cu bottom electrode on SiO_2_/Si, followed by deposition of the Pt top electrode. After an electroforming process^43,44^, a good resistive switching behavior has been observed (Fig. 5d). The switching from a low resistance state (LRS) to a high resistance state (HRS) occurs at a negative voltage −1.4 V, and vice versa at a positive voltage 1.7 V, with a resistance ratio at ~10^2^. It is a kind of bi-polar switching behavior^44^, with substantially low switching voltages, compatible with existing complementary metal-oxide-semiconductor (CMOS) integrated transistors. Such merits make it a good candidate for low power and high density device applications.

### Mechanism

The above results are based on the analysis of a sample with Pt and W both at 100 nm before the selenization process, from which a WSe_2_ film ~365 nm is finally obtained. Through tuning the W and Pt thicknesses, good crystallinity WSe_2_ films with different thicknesses from a few nanometers to the micrometer range have also been fabricated (see Supplementary Figs S6 and S7 for more details). We also verify the thickness relationship between the as-deposited W film and selenized WSe_2_ films (Fig. S7d). In this section, we will discuss the underlying mechanism involved in the epitaxial growth process of WSe_2_.

**Figure 6:**
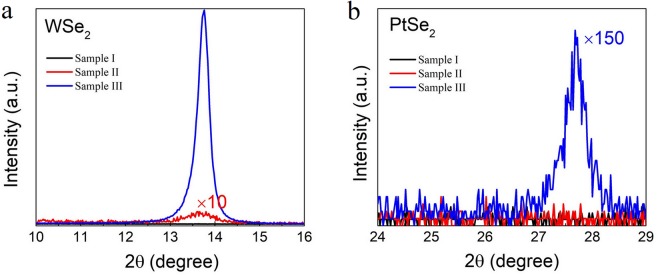
XRD patterns of the samples (I to III) grown at different conditions. (**a**) WSe_2_. (**b**) PtSe_2_. Sample I is grown at the selenization temperature of 350 °C. Sample II is firstly heated at 350 °C, and then increased to 450 °C. Sample III is firstly heated at 350 °C and then increased to 550 °C.

**Figure 7:**
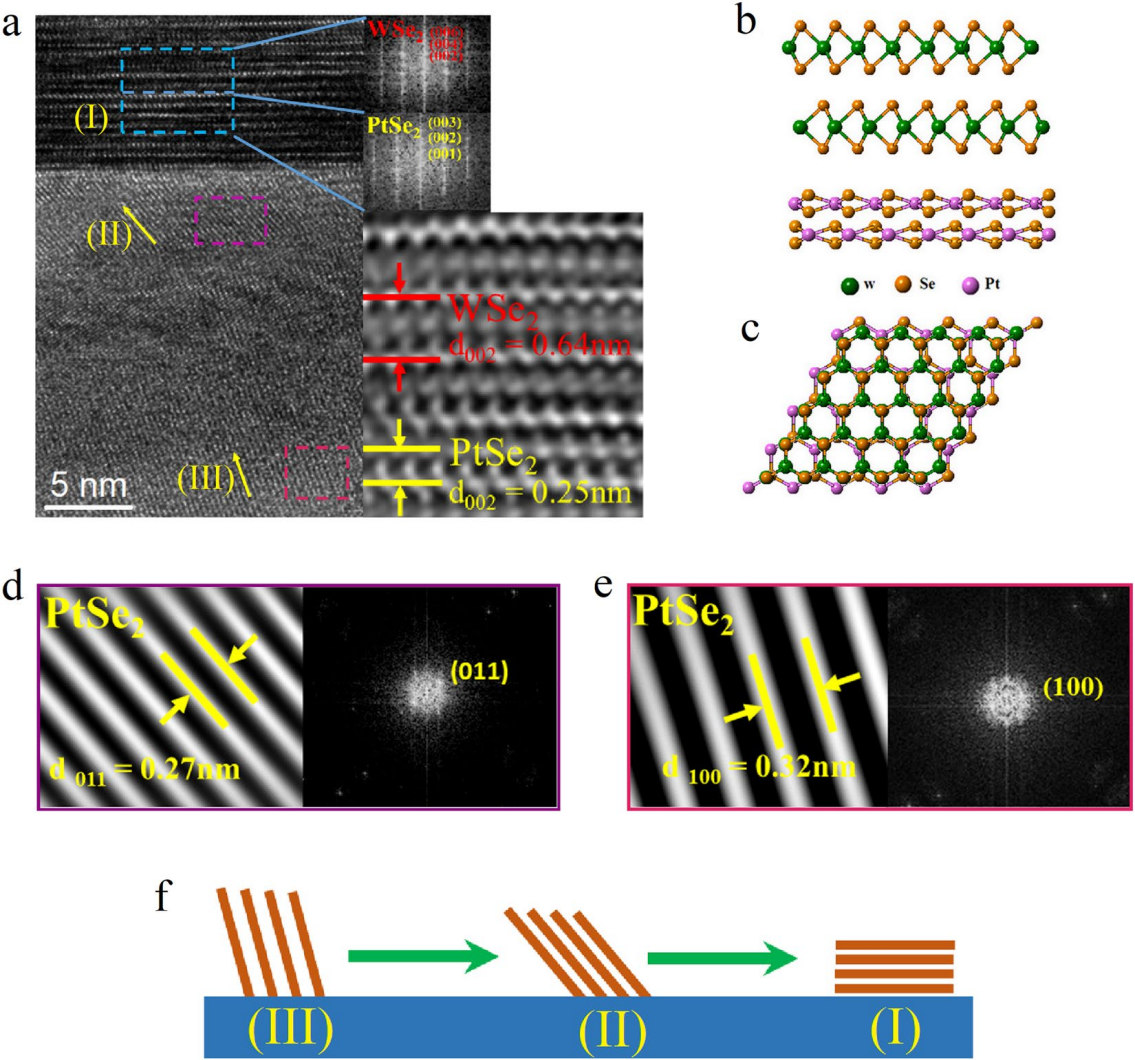
The growth of WSe_2_ assisted by the formation of a PtSe_2_ buffer layer. (**a**) Cross-sectional TEM image around the top interface. Right is a higher magnification of the cyan-color marked area, and the corresponding FFT patterns for the upper and lower regions, respectively. (**b**,**c**) Schematic illustration of the atomic structure of the WSe_2_/PtSe_2_ heterostructure: side-view and top view. The van der Waals epitaxial growth mode. (**d**) A higher magnification of the area near the top interface - the purple-color marked area in (**a**) (region II), and the corresponding FFT pattern. (**e**) The magnified image and the corresponding FFT pattern of the pink-color marked area in (**a**) (region III). (**f**) Schematic illustration of the growth process of PtSe_2_.

Figure 6 shows the XRD patterns of the samples by changing the growth conditions. Sample I is grown at the selenization temperature of 350 °C, and neither WSe_2_ or PtSe_2_ peaks are shown. Sample II is firstly heated at 350 °C, then increased to 450 °C. At this condition, WSe_x_ (x < 2) starts to appear but the growth rate is very slow. Sample III is firstly heated at 350 °C and then increased to 550 °C which is described in the first part. The XRD image for PtSe_2_ in Fig. 6b is the magnification of that shown in Fig. 1, for which a PtSe_2_ [001] peak can be clearly identified, along with the strong WSe_2_ [001] peak shown in Fig. 6a (Sample III). On the other hand, the growth of WSe_2_ directly on SiO_2_/Si without a Pt underlayer reveals poor crystalline quality (Fig. S3a). These results imply that PtSe_2_ could play an important role in promotion of the growth of WSe_2_. To understand this, we have further conducted cross-sectional TEM studies to confirm the atomic structure of the stacked film structure. Figure 7a shows the TEM image close to the top interface. Interestingly, a highly ordered PtSe_2_ buffer layer is found self-assembled below WSe_2_, compared to the complicated disordered interlayer. The FFT pattern shows a hexagonal lattice structure for both WSe_2_ and PtSe_2_ along the [001] direction, with a d-spacing of 6.4 Å and 2.5 Å respectively, consistent with the report by Wang *et al*.^45^. To demonstrate the growth scheme, the atomic geometry of the composite system is shown in Fig. 7b,c. As reported, both WSe_2_ and PtSe_2_ have a hexagonal structure with lattice constants a = b = 0.328 nm for WSe_2_ and a = b = 0.373 nm for PtSe_2_ respectively^45,46^. Even with a similar hexagonal atomic structure, a large in-plane lattice mismatch of 12.1% exists between two (001) planes. The growth mode is attributed to the so-called van der Waals epitaxial growth^1,47^, in contrast to the traditional epitaxial growth mode which requires a good lattice matching between two crystals. Epitaxial heterostructures by weak van der Waals interactions have been successfully realized for various 2D material systems in recent years^1–4^.

The region below the highly epitaxial WSe_2_/PtSe_2_ layers is a highly disordered interlayer. To further investigate the role of this layer, a detailed TEM analysis is also performed. It shows that complex phases are formed in the interlayer. Figure 7d shows the magnified image for the marked area (purple, region II, Fig. 7a) close to the top interface, where the HRTEM image and the corresponding FFT pattern reveal a PtSe_2_ lattice structure, with the (011) orientation. Compared to the above continuous PtSe_2_ layers (lower cyan-color marked area), the PtSe_2_ in the interlayer is more like a nano-crystalline structure surrounded by other phases. In the interlayer away from the interfaces, a highly disordered zone is formed, indicating complicated thermal diffusion dynamics during the growth process. Moreover, the crystalline PtSe_2_ away from the top interface is also investigated. Figure 7e illustrates the magnified image of the pink-color marked area in Fig. 7a (region III), and a PtSe_2_ lattice structure with the (100) orientation is shown. Different crystal orientations lead to different surface energies. In crystal growth, the mobile species tend to seek out low-energy positions and the equilibrium crystal states. The calculation of MoS_2_ shows that (001) plane has a much lower surface energy than the other directions^48^. With a similar structure to MoS_2_, it is reasonable to infer that the (001) plane for PtSe_2_ also has the lowest surface energy. As expected from the growth kinetics, the preferred growth orientation follows the crystalline plane which has the lowest energy. The simplified thermodynamic model for PtSe_2_ grown in the interlayer on the basis of the surface energy is shown in Fig. 7f, similar to the vertical to horizontal growth kinetics as reported^48^. Following the route to lower the energies, the highly ordered (001) PtSe_2_ buffer layer with the lowest surface energy is formed on the top of the disordered interlayer. The self-assembled (001) PtSe_2_ buffer layer then promotes the formation of (001) WSe_2_ epitaxial film through van der Waals epitaxial growth. Besides PtSe_2_, Pt_2_W has also been found at the interlayer region close to the bottom interface, for which we believe that the Pt-W alloy could come from the high temperature reaction process (see Supplementary Fig. S8 for more details). Through reducing the initial Pt thickness or increasing the selenization time, such alloy can be prevented (see Supplementary Figs S6 and S9 for details). Therefore, we believe that the presence of Pt_2_W is not critical to the growth of WSe_2_.

In parallel with the W 100 nm/Pt 100 nm sample, we also did cross-sectional HRTEM analysis of a thinner sample with the initial thicknesses of W 5 nm/Pt 2 nm. A PtSe_2_ buffer layer has also been observed after selenization (see Supplementary Fig. S10 for more details). With help from the Pt underlayer, great advantages have been established, compared to the direct growth of WSe_2_ on SiO_2_/Si. The balance between surface energy and interface energy as well as the balance between energetics and kinetics, controls the growth process. The disordered interlayer region comes from complex reactions during the high temperature selenization process. Although the growth kinetics need to be further investigated for the interlayer region, we suggest that this interlayer between the highly epitaxial WSe_2_/PtSe_2_ layers and the substrate might behave as a buffer to accommodate the complicated balance, leading to the formation of the self-assembled PtSe_2_, which finally promotes the epitaxial growth of WSe_2_.

Large surface energy, poor wettability and the large lattice mismatch between a TMDC material and an insulating substrate are three common challenges in achieving high quality crystalline films^20^. Direct growth of large area WSe_2_ as well as other TMDC materials on SiO_2_/Si has been proved to be very difficult. The introduction of a buffer layer could be the choice to overcome such difficulties. Recently, growth of high quality graphene on SiO_2_/Si with a boron nitride buffer layer has been reported^49^. Analogous to a buffer layer in conventional epitaxy^50^, the mechanism and the method proposed here has a similar advantage, and could be applied to the growth of other TMDC materials.

## Conclusion

In summary, we have reported an example of epitaxial growth of WSe_2_ on inch-sized SiO_2_/Si using a two-step selenization method. Crystalline WSe_2_ thin films with high uniformity have been obtained, which shows great potential of this method for large-scale fabrications. The existence of the self-assembled PtSe_2_ buffer layer promotes the (001) WSe_2_ growth through the van der Waals epitaxial growth mode. Based on the fabricated thin films, high performance memory devices have also been demonstrated. Although the detailed underlying growth mechanisms need to be further explored, the method and the strategy described here may be applicable to the preparation of other TMDC materials in a large scale mode at a much lower temperature, and open up new possibilities in 2D materials engineering and applications.

## Methods

### Epitaxial WSe_2_ growth

Substrates cut from silicon wafer with 300 nm thermally grown SiO_2_ on top were used for the growth. Prior to the Pt film deposition, a 3 nm Ta was deposited as the adhesion layer, by magnetron sputtering. Then W with variable thicknesses was deposited by another sputtering process. α-W(110)/Pt(111) was achieved after the process. Then the α-W(110)/Pt(111)/Ta/SiO_2_/Si substrate was placed at the center of a tubular furnace (4-inch-diameter tube). Selenium ingots (0.3 g) were placed in a separate ceramic boat, 6.5 cm away from the sample. The Se vapor was delivered by a N_2_ flowing gas. The sample was first heated to 350 °C and annealed for 30 min, followed by the final treatment at 550 °C for about 12 seconds. Finally, the furnace was naturally cooled to room temperature.

### Characterization

The crystallinity of the samples was determined by X-ray diffraction (XRD) using a diffractometer with Cu Kα radiation (Shimadzu LabX XRD-6000). Grazing-incidence XRD (GIXRD) measurements were performed with D8 Discover with GADDS (Bruker AXS Gmbh, Karisruhe, Germany). Cross-sectional transmission electron microscopy (TEM) images were taken with a FEI Tecnai Osiris system and top-view TEM images were taken with a FEI Tecnai F20 system. The morphologies of the prepared samples were obtained using a field emission scanning electron microscopy (SEM) (Hitachi, S-8010). X-ray photoemission spectroscopy (XPS) was performed by a Ulvac-PHI 1600 spectrometer with monochromatic Al Kα X-ray radiation (1486.6 eV). Raman and photoluminescence (PL) spectroscopy were conducted with HORIBA LabRAM HR800 except for the Raman mapping. We used 632 nm excitation wavelength for the measurements. The Si peak at 520 cm^−1^ was used as a reference for wavenumber calibration. The Raman mapping was performed on Nanofinder 30 (Tokyo Instruments, Inc.).

## Supplementary information

Supplementary Information
