# Supplementary material for: Scalable Epitaxial Growth of WSe2 Thin Films on SiO2/Si via a Self-Assembled PtSe2 Buffer Layer
Source: Sci Rep. 2019 May 29;9:8017. doi: 10.1038/s41598-019-44518-3 (PMC6541630; doi:10.1038/s41598-019-44518-3)
Supplement: Supplementary file 1 — Supplementary Information [file 41598_2019_44518_MOESM1_ESM.pdf]

Supporting Information

Scalable Epitaxial Growth of WSe_2 Thin Films on SiO_2/Si *via* a Self-Assembled $PtSe_2$ Buffer Layer

Pei-Chen Wu, Chun-Liang Yang, Yuanmin Du*, and Chih-Huang Lai*

Department of Materials Science and Engineering, National Tsing Hua University, Hsinchu 30013, Taiwan

Correspondence and requests for materials should be addressed to Y.M.D. (email: ynmin.du@gmail.com) or C.H.L. (email: chlai@mx.nthu.edu.tw)

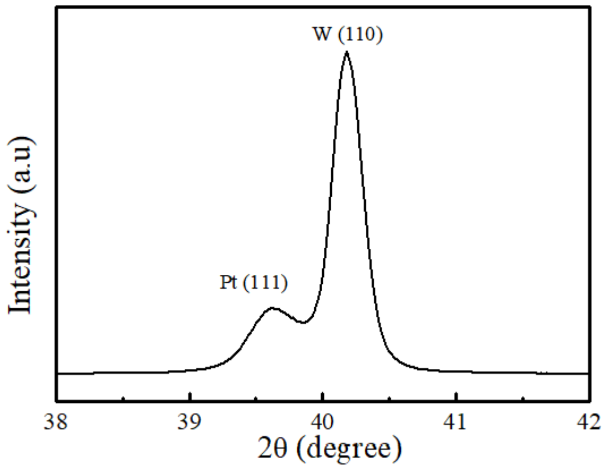

Figure S1. XRD pattern of the as-deposited W(100 nm)/Pt(100 nm)/Ta (5 nm)/SiO_2/Si before the high temperature selenization process. Using Ta as the adhesion layer, the growth of Pt(111) has been achieved. A α-phase W(110) film is formed at the subsequent sputtering process.

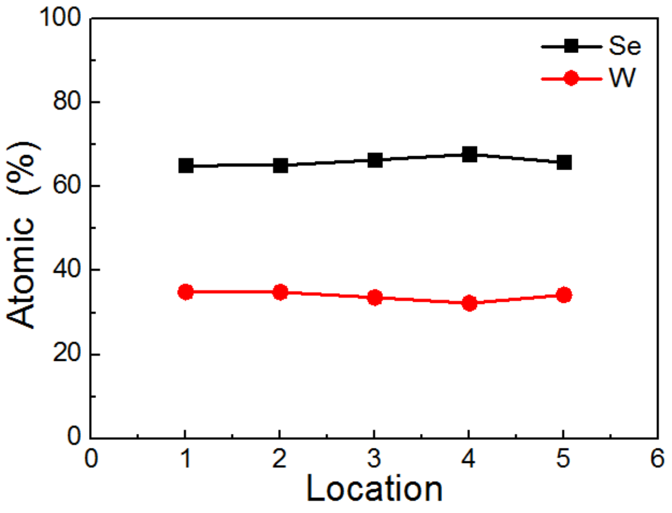

Figure S2. Composition scan at different locations of the WSe_2 layer obtained by EDX spectroscopy.

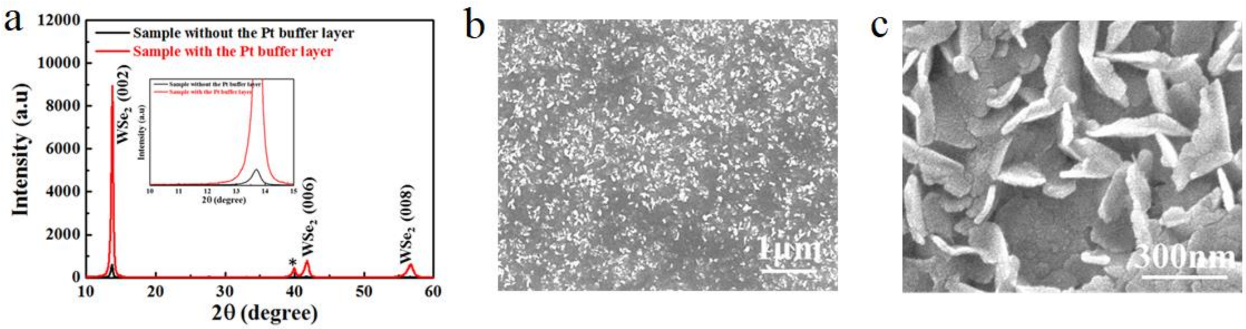

Figure S3. (a) XRD patterns of the WSe_2 films with and without the Pt underlayer, with W the same thickness (100 nm). The inset is the enlarged XRD patterns around the WSe_2 (002) range for both samples. (b) and (c) Top-view SEM images for the sample without the Pt underlayer.

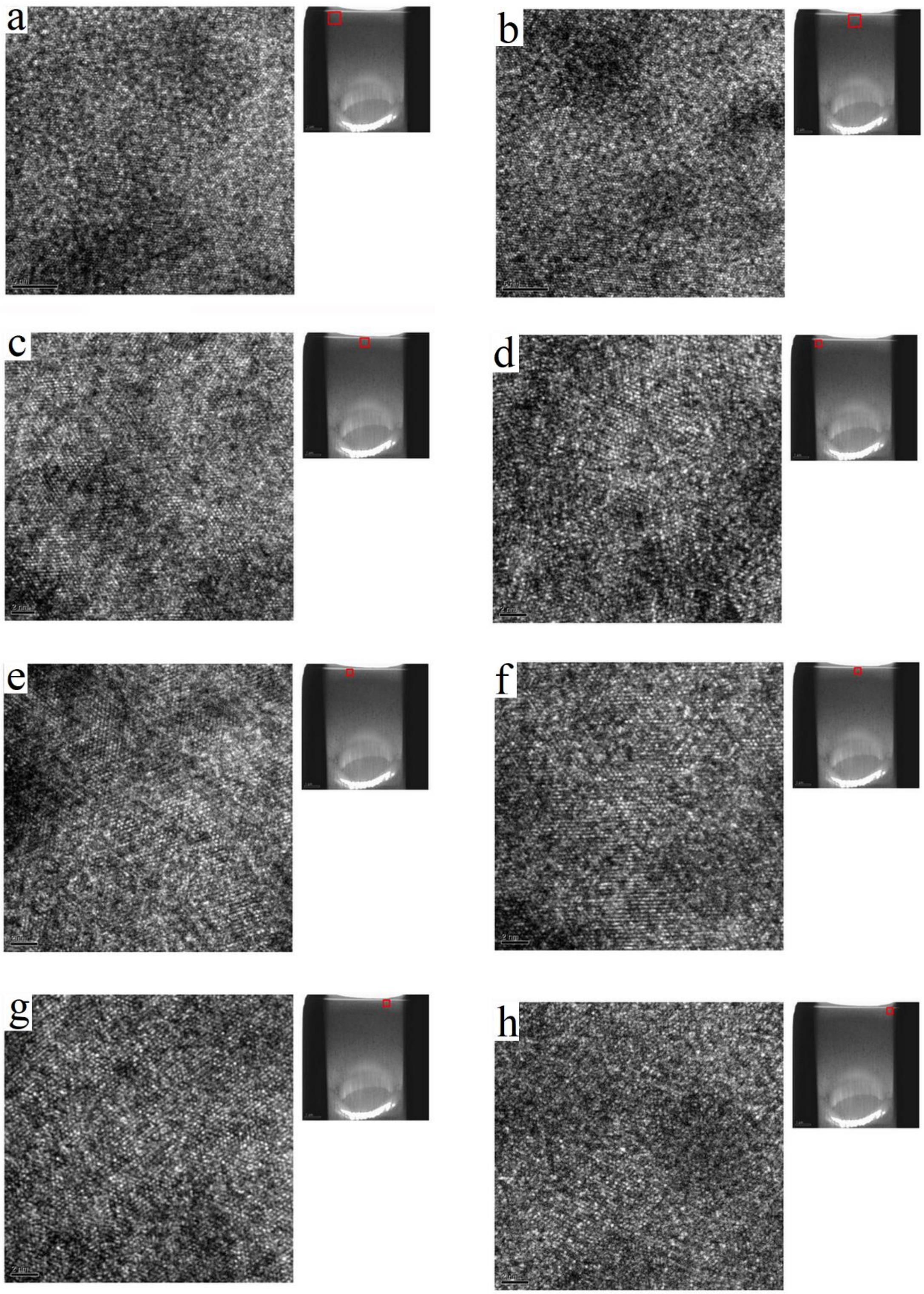

Figure S4. (a) to (h) Top-view STEM images for different locations of the TEM sample after a FIB cut. Left: TEM image; right: the location (red squared).

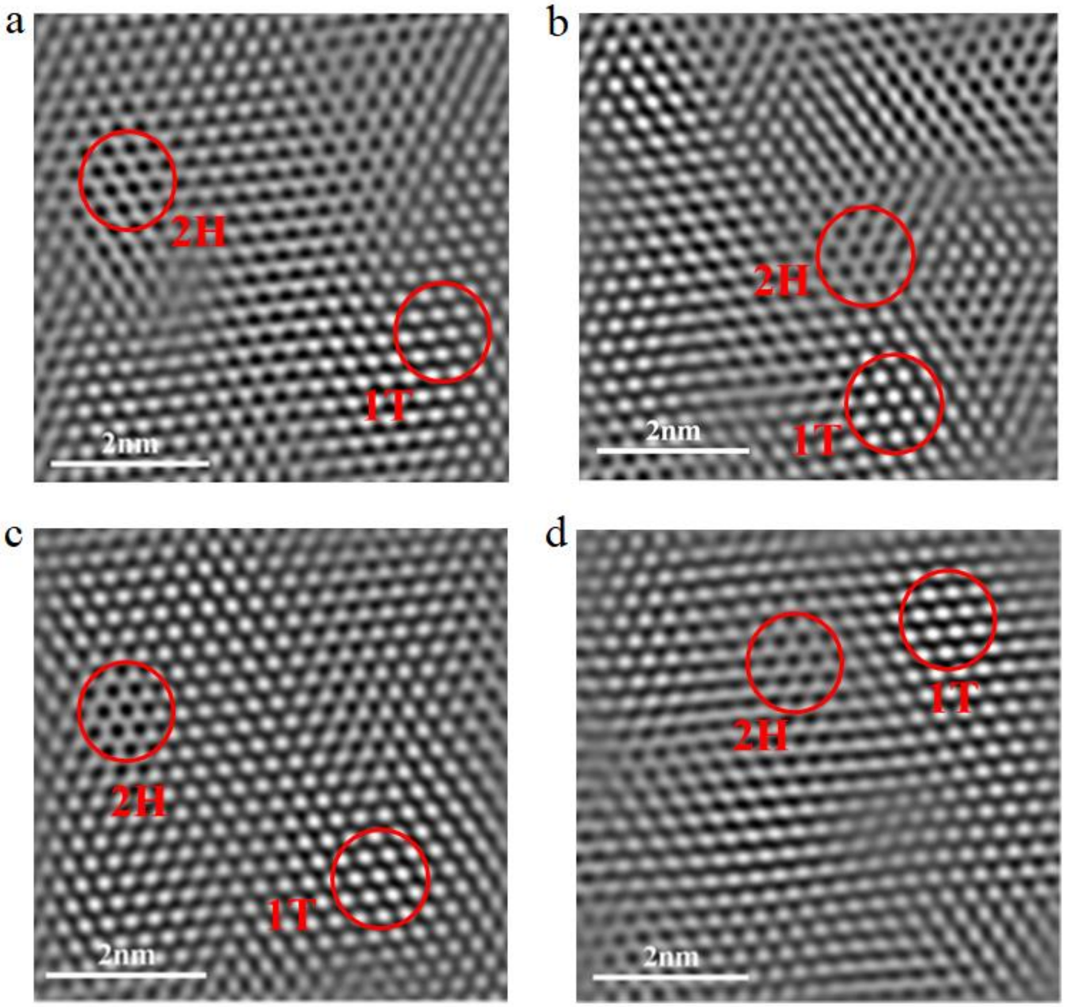

Figure S5. (a) to (d) HAADF-STEM images showing the coexistence of 2H and 1T hexagonal structures of WSe_2.

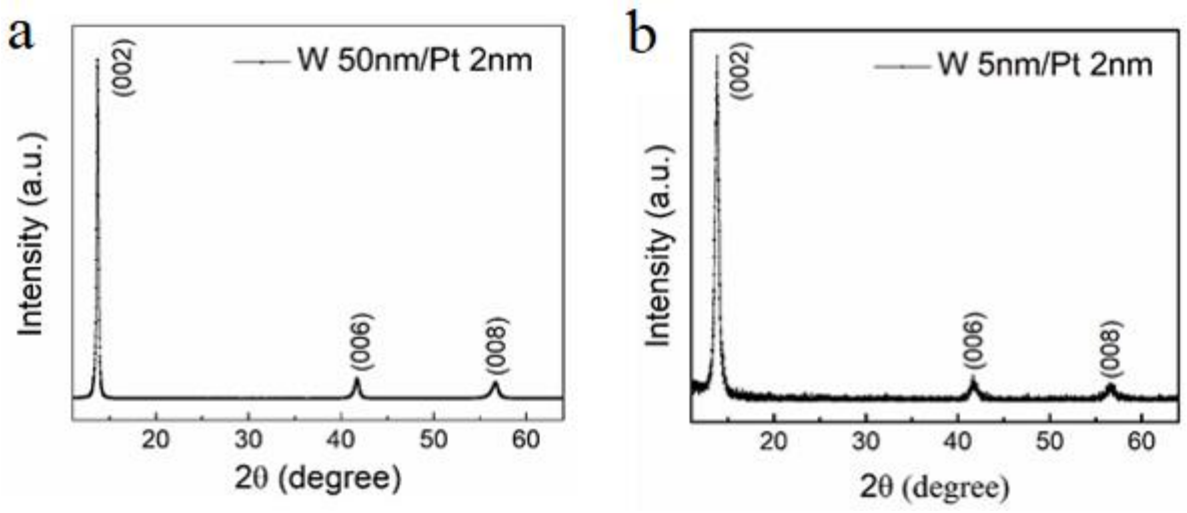

Figure S6. XRD patterns of the samples with different initial W and Pt thicknesses (thin samples). (a) W 50 nm/Pt 2nm. (b) W 5 nm/Pt 2 nm. Peaks related to different WSe_2 (00ℓ) planes are shown.

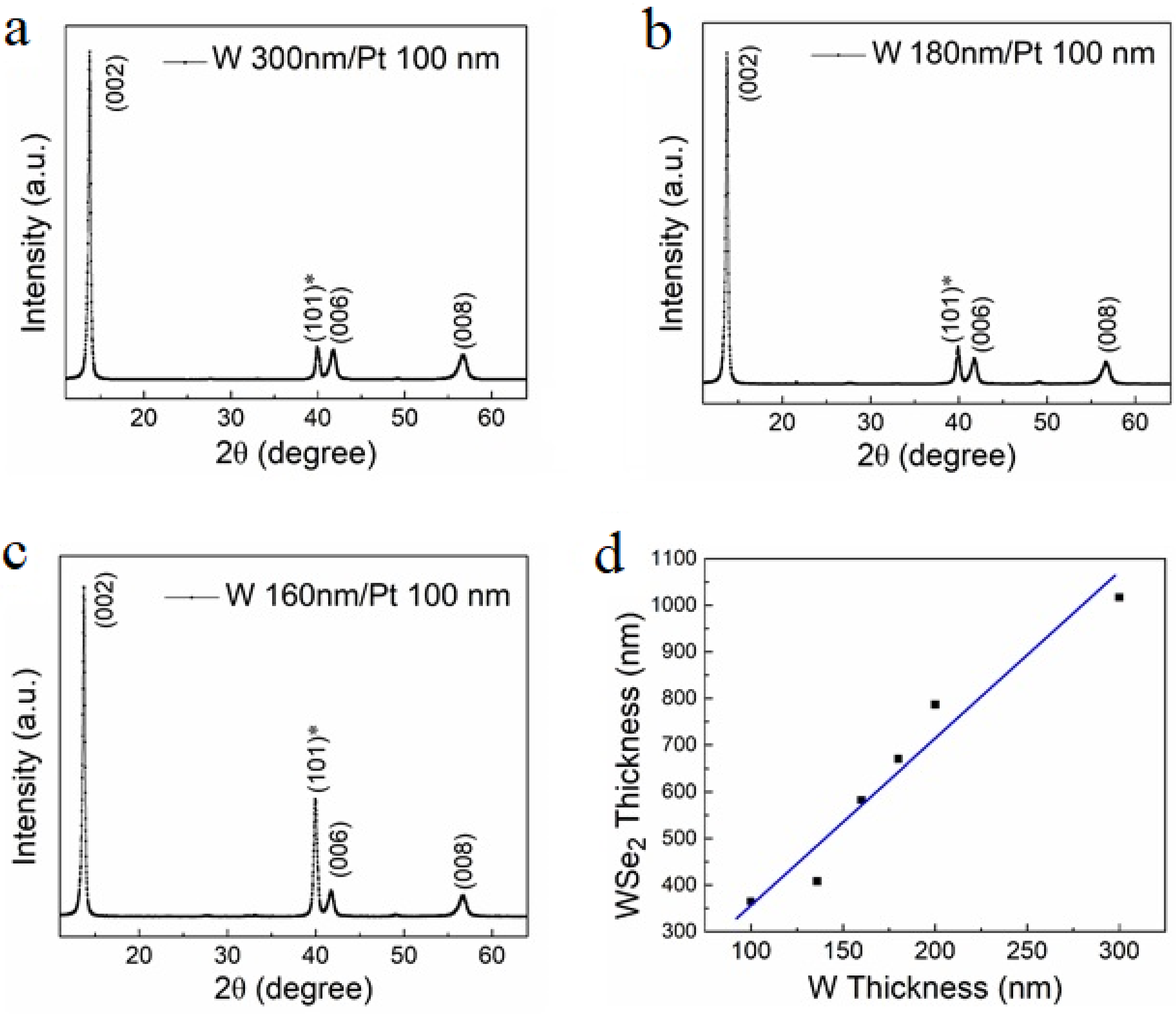

Figure S7. XRD patterns of the samples with different initial W and Pt thicknesses (thick samples): (a) W 300 nm/Pt 100nm; (b) W 180 nm/Pt 100nm; (c) 160nm/Pt 100nm. WSe_2 peaks related to (00ℓ) planes are shown. The peak $(101)^*$ is related to Pt_2W. (d) Thickness of WSe_2 *versus* W thickness.

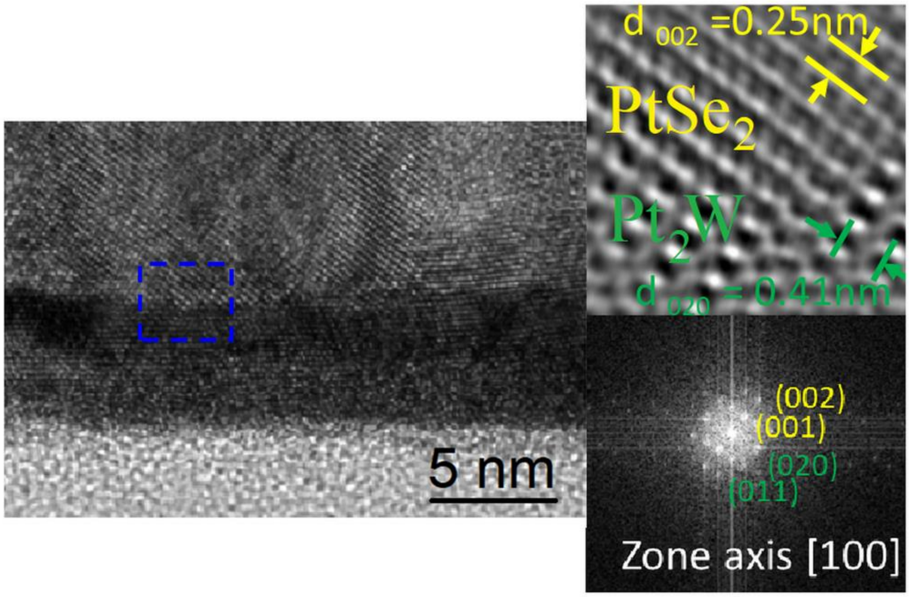

Figure S8. TEM image around the bottom interface of the interlayer region. Right shows the magnified image and the corresponding FFT pattern of the blue-color marked area.

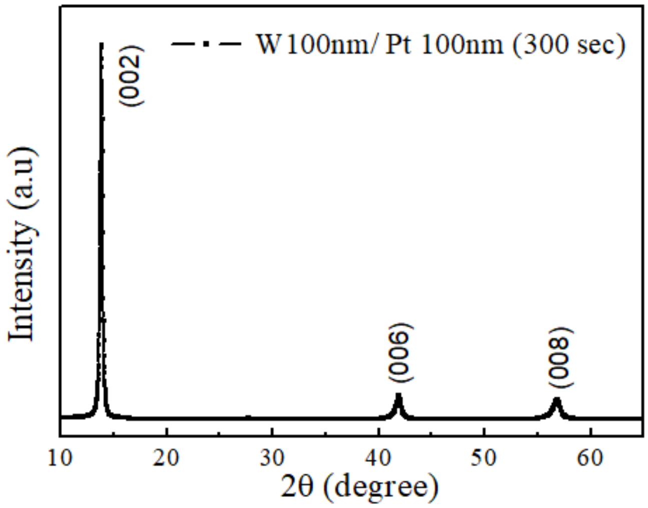

Figure S9. XRD pattern of the sample with initial W and Pt thicknesses at W 100 nm/Pt 100nm: first heated to 350 °C and annealed for 30 minutes, followed by the final treatment at 550 °C for about 300 seconds. WSe_2 peaks related to (00ℓ) planes are shown.

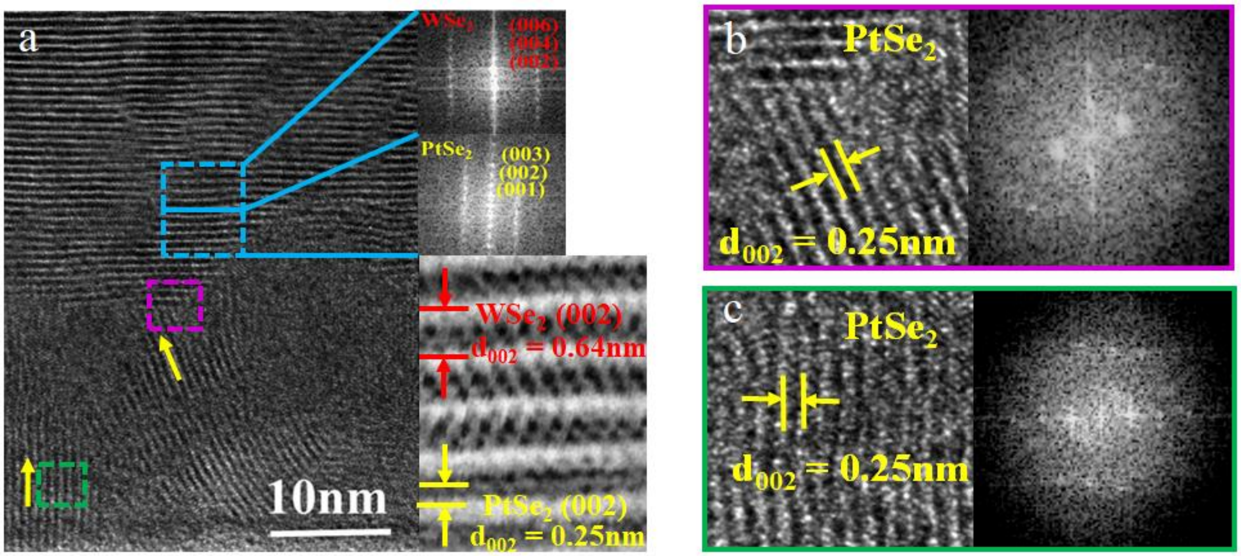

Figure S10. (a) Cross-sectional TEM image analysis for the sample with the initial thicknesses of W 5 nm/Pt 2 nm. Below WSe_2 is an ordered $PtSe_2$ buffer layer. Below the ordered $PtSe_2$ buffer layer is a region where rotation of $PtSe_2$ lattice has been shown. (b) A higher magnification of the purple-color marked area in (a), and the corresponding FFT pattern. (c) A higher magnification of the green-color marked area in (a), and the corresponding FFT pattern.
